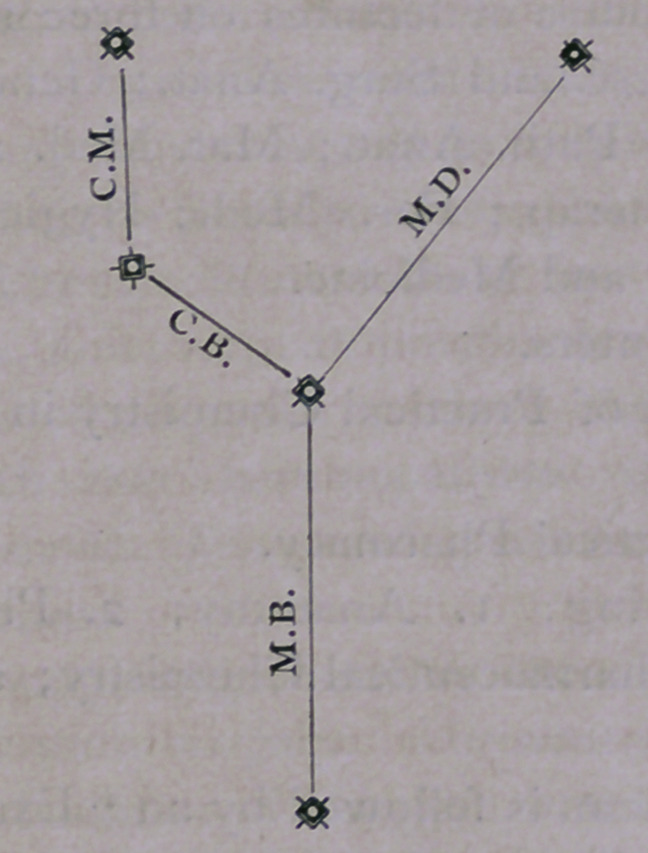# Foreign Correspondence

**Published:** 1871-03

**Authors:** J. H. Etheridge

**Affiliations:** Glasgow, Scotland


### Foreign Correspondence.

GLASGOW, Scotland, January.

EDITOR CHICAGO MEDICAL JOURNAL:

*Dear Sir*—Medical education at home, is very different from that in England, and with your concurrence, I would like to lay before your numerous readers something of the mode of "making doctors," as I saw it in London.

The first hospital I visited, was St. Bartholomew's, and as I passed through the gate I inquired for some one that would give me information concerning lectures, clinics, and ward-visiting, and the porter directed me to *Mr.* Marant Baker's door. I found his door and was soon ushered into his presence, at first supposing he was secretary or some such functionary, because he was called *Mr.* instead of *Dr.* Baker. Imagine my surprise, to learn, subsequently, that he is one of the assistant surgeons of that hospital —has a ward in it as Professor of Physiology and Pathological Anatomy in the school connected with St. Bartholomew's, and has revised some work on physiology, and yet is called *Mr.* Baker! I looked over the list of the medical faculty for his school and found some of the gentlemen's names preceded by *Mr.* and the balance by *Dr.* At home, all faculty names have the Dr. before or the M.D. after each one.

This new idea led me to inquire "why the difference?" and the answer "In England, all *Physicians* are called *Doctors*, and all *Surgeons* are called *Misters* (properly *Masters*)" determined me to look up the subject of medical education more fully, and the results I herewith transmit: I *may* make some slight misstatements, but in the main I think you will find me correct.

In the United States, physicians and surgeons are educated together from their first readings till their graduations. In England, they are in the same classes only a portion of the time; beyond that time they may be said to be separately trained. All surgeons' signs and doorplates have the name preceded by Mr. and followed by "Surgeon"—*e. g.*, "Mr. J. H. Smith, Surgeon;" Prof. Erichsen's doorplate reads simply, "*Mr. Erichsen.*" If a man be both physician and surgeon, then he has it "Dr. ——, Surgeon."

Four years' study and hospital walking, followed by a satisfactory final examination, give a young man the degree "*Bachelor of Medicine.*" Both physicians and surgeons must take this degree, and they remain in the same classes, attend same lectures, clinics and wards, till this degree is obtained. Subsequently, the physician can take his M.D. degree, by two years' attendance upon hospital work, and a required examination, thus consuming *six years* in acquiring a degree, that, at home, is to be had by three years' work. The surgeon, after obtaining the M.B. degree, must take the *Bachelor of Chirurgery* degree, and this is to be followed by two years in surgical wards in hospitals before he can take his *Master of Chirurgery* degree; when he has that degree, then he is "Surgeon," and not till then.

Finding out these points revealed to me, how it is, that six years must be spent in acquiring a physician's or surgeon's degree. Very many of the profession never go any farther than the M.B. degree, for with that they can practice medicine and collect fees in any of the British possessions. Ambitious men, however, get the M.D. or C.M. degree and run the gauntlet at the Royal College of Surgeons and Physicians, and at Apothecaries Hall for the Members' and Fellows' honors there. The following diagram may serve to illustrate the two paths pursued by M.D.'s and C.M.'s:

Both occupy six years. The principal part of studying comes in the first four years, and I have been at some pains to so arrange it, that it can be easily seen, what a young man has to go through with, in order to get even his first degree, M.B.

Before beginning his studies, the young man must see that his name is entered on the books of the Medical Registrar, giving notice where he intends to study and attend lectures. Four times annually must he report to the registrar, give an account of himself, and see that his name is

"entered" on the books. It is for the student's own interest to see to this registration business, otherwise, that functionary's certificate not giving enough time to the young man's studies to suit his examiners, will not admit him to the examinations, and he must wait over long enough to make up for lost time with the registrar reporting to him quarterly.

The M.B. examination is divided into two parts, viz.: "*First* and *Second* M.B. Examinations," and they are two years apart. The first requisite to the *first* examination, is a degree in *arts* at some university—and if the required piece of vellum cannot be produced, then the young man is at once examined in the branches necessary—Greek, Latin, mathematics, natural philosophy, elementary chemistry, English language and literature, history, and one modern language, German or French. This examination must be passed immediately after registering. In one year from that time comes in another examination, called the "Preliminary Scientific Examination," and consists of a close questioning in the sciences—chemistry, botany, optics, etc. Then follows another year of professional studies and hospital walking, following which, is the first M.B. Examination, and here the student must produce certificates to the following effect:

(a) Of having completed his nineteenth year.

(b) Of having passed Preliminary Scientific Examination.

(c) Of having attended a full course of lectures on three subjects from the following list: Desc. and Surg. Anat.; General Anat. and Physiol.; Comp. Anat.; Path. Anat.; Mat. Med. and Pharm.; General Path.; Gen. Therap.; For. Med.; Hygiene; Midwif. and Dis. Children; Surg.; and Medicine.

(d) Of having dissected, two winters.

(e) Of having attended a course of Practical Chemistry in the laboratory.

(f) Of having attended to Practical Pharmacy.

And he is examined in the following: 1. Anatomy; 2. Physiology; 3. Materia Medica and Pharmaceutical Chemistry; and 4. Organic Chemistry.

This examination, like most of them, is followed by an "Examination for Honors," and the candidate who evinces sufficient merit, in the estimation of the examiners, in one or more of the above four subjects, receives a prize of forty pounds ($200) annually for the subsequent two years, in quarterly installments.

In two years from the first M.B. examination, comes the second, and no candidate shall be admitted thereto, unless he have produced certificates to the following effect:

(a) Of having passed the first M.B. Examination.

(b) Of having, subsequently to the first M.B. Examination, attended lectures on two subjects from the preceding list, not previously attended.

(c) Of having conducted *twenty* cases of labor.

(d) Of having attended for two years, surgical practice of a hospital, with clinical instruction and lectures on clinical surgery.

(e) Of having attended medical practice of a hospital two years, with clinical instruction and lectures in clinical medicine.

(f) Of having subsequently attended to practical medicine, surgery or midwifery, with special charge of patients in a hospital, infirmary or dispensary, for six months.

(g) Of having attained proficiency in vaccination.

The candidate is examined in the following branches:

1. General Path.; General Therapeutics and Hygiene. 2. Surgery. 3. Medicine. 4. Midwifery. 5. Forensic Med. 6. Medical and Surgical Anatomy. 7. Path. Anatomy. 8. Path. Chem.

After this examination also comes one for honors, and the successful candidate receives $250 annually for two years. Here the young man may stop in hospital work, and "locate" and practice so long as he lives—*never* be an M.D., and yet practice medicine and be upheld in courts of law and compel patrons to pay fees. No one, however, can practice here, be he anything whatsoever in the line of a quack, without the Bachelor of Medicine degree. I learn that homœopathists throughout England are *properly* educated, *first*, and preserve their regular diplomas for emergencies.

Supposing, however, the candidate wishes to continue work and study surgery. His next step will be to pass the examination for Bachelor of Surgery, and if he can meet the requisitions, he can present himself for this trial within three weeks after having passed his second M.B. examination. He must first present certificates to the following effect:

(a) Of having taken the degree of Bachelor of Medicine.

(b) Of having attended a course of instruction in operative surgery, and of having operated on the dead subject.

Passing this examination makes the candidate a Bachelor of Surgery, and in two years thereafter, he can present himself for the final gauntlet, and must present certificates to the following effect:

(a) Of having taken the B.S. degree.

(b) Of having, thereafter, attended for two years, to clinical or practical surgery, in a hospital or medical institution.

(c) Of moral character, signed by two persons of respectability.

He must pass examination on the following:

1. Logic and moral philosophy; names, notion and propositions; syllogism, induction and subsidiary operations; the intellect; the senses; the will, including the theory of moral obligation.

2. Surgery.

If a young man wishes to pursue medicine instead of surgery, he begins work with that point in view, soon as he has become Bachelor of Medicine, and the requisites to obtaining the degree of M.D. are, *two* years in a hospital, attending to clinical or practical medicine, followed by an examination the same as the candidate for the Master of Surgery degree passes, with the exception of substituting *Medicine* for *Surgery* in the items of questioning.

The first thing that greatly impressed me in learning the above facts, was, the exceedingly large proportion of hospital work that is necessary to obtaining *any* degree. *Nine-tenths* of the studies are inside the wards and amphitheatres.

Almost every school is connected with some hospital, and the didactic lectures are delivered within the hospital walls. Students here go from the lecture-room to the ward or amphitheatre without putting their caps on even, in many schools following their teacher, and seeing, on the patient, what the lecture has prepared them for. Post mortems are witnessed at any time without going a half mile for the privilege. In Guy's, students—*each one*—must, among other certificates, bring forward one to the effect, that he has made the post mortems of the hospital under the direction of the demonstrator of anatomy, for the space of *three months.*

All operations, lectures and clinics for the day, are advertised on the "board" hanging up by the outside entrance, and almost daily, some operation is performed. On one day, I saw at one sitting, Sir Henry Thompson cut for stone in one patient, crush a stone in another, Prof. Erichsen remove two cancers from breasts, and Prof. Jno. Marshall operate on a necrosed tibia, in the operating amphitheatre at University College Hospital.

The method of studying physiology, as I saw it in the Univ. Coll. Hosp. school, charmed me. I find it is entirely the fashion in all the English and Scotch schools to study this branch similarly, viz.: in *laboratories*, practically, just as chemistry and pharmacy are studied. It is not compulsory, to pursue it thus—it is wholly optional, but nearly all have the good sense to avail themselves of facilities thus offered. Students pursuing physiology in the laboratory are supposed to know anatomy well beforehand, and are thus prepared to, intelligently, take the first step in this work, viz., use the microscope. Every tissue is carefully examined, and the eye so trained, that future examinations reveal no guesswork on the part of students. The mechanism of microscopes is *first* taught by Jno. Hughes Bennet in his laboratory, and *then* the *tissues*. The instruments are furnished by the school, and magnify from fifty to five hundred diameters. Subsequently, is taught practical experimental philosophy, including experiments on animal electricity, contractility, excitability, pulsation, vision, voice, heat, respiration, etc., etc., with the aid of modern instruments, such as the galvanometer, kymographion, sphygmograph, myographion, ophthalmometer, laryngoscope, thermometer, spectroscope, etc.; analysis of morbid and healthy urine, of blood, bile, bone, etc., are carefully taught.

For purposes of investigation and experiment, guinea pigs and frogs are kept constantly on hand. The former are generally kept in great numbers, being bred and kept on the school premises, by most schools. Frogs are imported from Germany, English specimens being too small.

The private laboratory of the professor is a perfect forest of small and large bottles and jars, containing tissues preserved in alcohol, of all parts of the body, human and animal.

The dissecting room is generally a very quiet place, no talking by students being allowed, except with the professor or demonstrators; of the latter, there are usually not less than four, and work can be carried on with little or no interruption by waiting for some one to assist the students. I had a particular desire to see the room, in Edinburgh, wherein the female students had so fearfully shocked the young Scotchmen, by dissecting the genitals in their presence, and caused thereby such an overflow of virtuous indignation and rude behavior to be shown by those young lords of creation. I accordingly went to it and found about sixty men at work so quietly, that one would think them all dumb. They were in two rooms, aggregating in area about the same as Rush College dissecting room; Prof. Turner and his assistants were among the students, showing and directing them. On the walls are hung "Rules for the dissecting room," notifying the students that a limited number of days is all they can have to dissect each subject in, *e. g.*, four days the subject will be kept on the back, before opening the chest and abdomen; two days allowed for examining viscera; three days with subject on face; two days for position for lithotomy; so many days for something else; consuming about fourteen or sixteen days in all. The cadaver is *turned* at the day appointed, per rules, whether it is dissected or not, and at the end of the fourteen or sixteen days the table is cleared.

During the first four years of professional studies, the classes are "graded." Studies—certain ones are "recommended" by the faculty for each year and these "recommendations" are the means of so thoroughly dividing first year from third year students, that the former are rarely seen in lectures on subjects "recommended" for the latter. No lecturer has at any one time, over two hundred and fifty students in the school at Edinburgh, where there are over *six hundred* students in all.

Medical books in London are not cheaper than at home, some of them cost *more*. We have to pay here in gold, and at home most of the books can be had for as many dollars and cents in paper currency. Nearly every man connected with a hospital, whether a professor or assistant, has edited or revised some work, and the endless array of professional works one sees in publishing houses and bookstores exceeds anything that ever I saw in that line. Surgical instruments cost more here than in the United States.

Medical students here, as a rule, are in better circumstances, financially, than those at home. Remaining in hospitals so much of the four years as they are required, necessarily takes a good deal of money, and a poor man cannot afford it. A large proportion of the students take their degree in arts at some college and can easily afford it. Upon the whole, fees are higher than in the United States. *Everything* costs more here in medical schools; and physicians' and surgeons' fees are higher than with us. They charge a guinea ($5) for looking at a man.

Yours, etc., J. H. ETHERIDGE.

## Figures and Tables

**Figure f1:**